# Phototherapeutic effect of transformable peptides containing pheophorbide a on colorectal cancer

**DOI:** 10.1080/10717544.2022.2075987

**Published:** 2022-05-25

**Authors:** Zhiqin Zhang, Kaixin Wang, Manting Liu, Panxiang Hu, Yuchen Xu, Dongge Yin, Yuchang Yang, Xiaoxv Dong, Changhai Qu, Lu Zhang, Jian Ni, Xingbin Yin

**Affiliations:** a School of Chinese Material Medical, Beijing University of Chinese Medicine, Beijing, China; b Department of Biomedical Engineering, Southern University of Science and Technology, Shenzhen, China

**Keywords:** Phototherapy, self-assembled peptide, supramolecular nanofibrils, bioavailability improvement, photothermal conversion efficiency

## Abstract

Photodynamic therapy (PDT) and photothermal therapy (PTT) have attracted research interest for their noninvasive nature and selective treatment of tumor tissues. They are effective through the generation of reactive oxygen species (ROS) or heat. Nevertheless, several problems, including low bioavailability and long-lasting cutaneous photosensitivity, have limited their clinical application. In this study, we reported an in situ self-assembly strategy that could improve various biological properties of the photosensitizer *in vivo*. A photosensitizer connected to a receptor-mediated smart peptide can self-assemble into nanoparticles (NPs) under the force of hydrophobic interaction and then transform into a nanofibrillar network after attaching to the tumor cell surface with the help of the β-sheet-forming peptide KLVFF. The supramolecular structural changes deeply affected the PDT and PTT properties of the photosensitizer on tumors. After being aggregated into the nanostructure, the water solubility and targeting ability of the photosensitizer was ameliorated. Moreover, the improvement of the photothermal conversion efficiency, ROS generation, and tumor retention followed the formation of nanofibrils (NFs). This self-assembly strategy showed the ability of supramolecular nanofibrils to improve the bioavailability of photosensitizers, which provides a new potential treatment avenue for various cancer therapies.

## 1. Introduction

With the increase in unhealthy diets and more sedentary lifestyles, colorectal cancer incidence and mortality are growing rapidly worldwide, ranking third in terms of incidence and second in terms of mortality (Sung et al., 2021). Surgical resection is the primary treatment for colorectal cancer (Cubas et al., 2007), but one-third of patients still suffer a worsened clinical condition and recurrence (Johnston, 2005; Correale et al., 2006). Therefore, effective drugs are urgently needed to improve the prognosis of the disease. Phototherapy, including photodynamic therapy (PDT) and photothermal therapy (PTT) are attractive for colorectal cancer therapy, as they produce reactive oxygen species (ROS) or heat (He et al., 2016; Xu et al., 2017; Lan et al., 2018). Compared with chemotherapy and radiotherapy, phototherapy could achieve the effect of local treatment by controlling the position of the irradiation, firstly used for the clinical treatment of bladder cancer in 1993, and then clinically approved for the therapy of other cancers (Brown et al., 2004; Xodo et al., 2012; Li et al., 2013). However, the limitations of photosensitizers are nonignorable, including poor tumor targeting ability and water solubility, low photothermal conversion efficiency, as well as short tumor retention time, which hinder their clinical application (Felsher, 2003; Sun et al., 2009; Oh et al., 2013).

Previous studies have shown the advantages of self-assembled peptides in improving the bioavailability and targeting of photosensitizers in a controllable manner (Li et al., 2015, 2021; Chang et al., 2022). Under the driving force of noncovalent bonding (hydrophobic interaction, hydrogen bonding, π–π stacking) (Abbas et al., 2017; Qi et al., 2018), peptides can self-assemble into various nanomaterials, such as nanoparticles (Callmann et al., 2015), nanofibers (Zhang et al., 2021), and nanotubes (Zerda et al., 2012). These structural changes are associated with the sequence of amino acids and environmental parameters (Cui et al., 2014; Frederix et al., 2015; Cao et al., 2017). Self-assembled peptides can not only create new functions by their building blocks (Qiao et al., 2017), but also integrate various properties by the incorporation of functional molecules to fulfill the specific demands of therapy, which has aroused intense interest among researchers (Lindgren et al., 2006; Zhang et al., 2021).

**Scheme 1.**
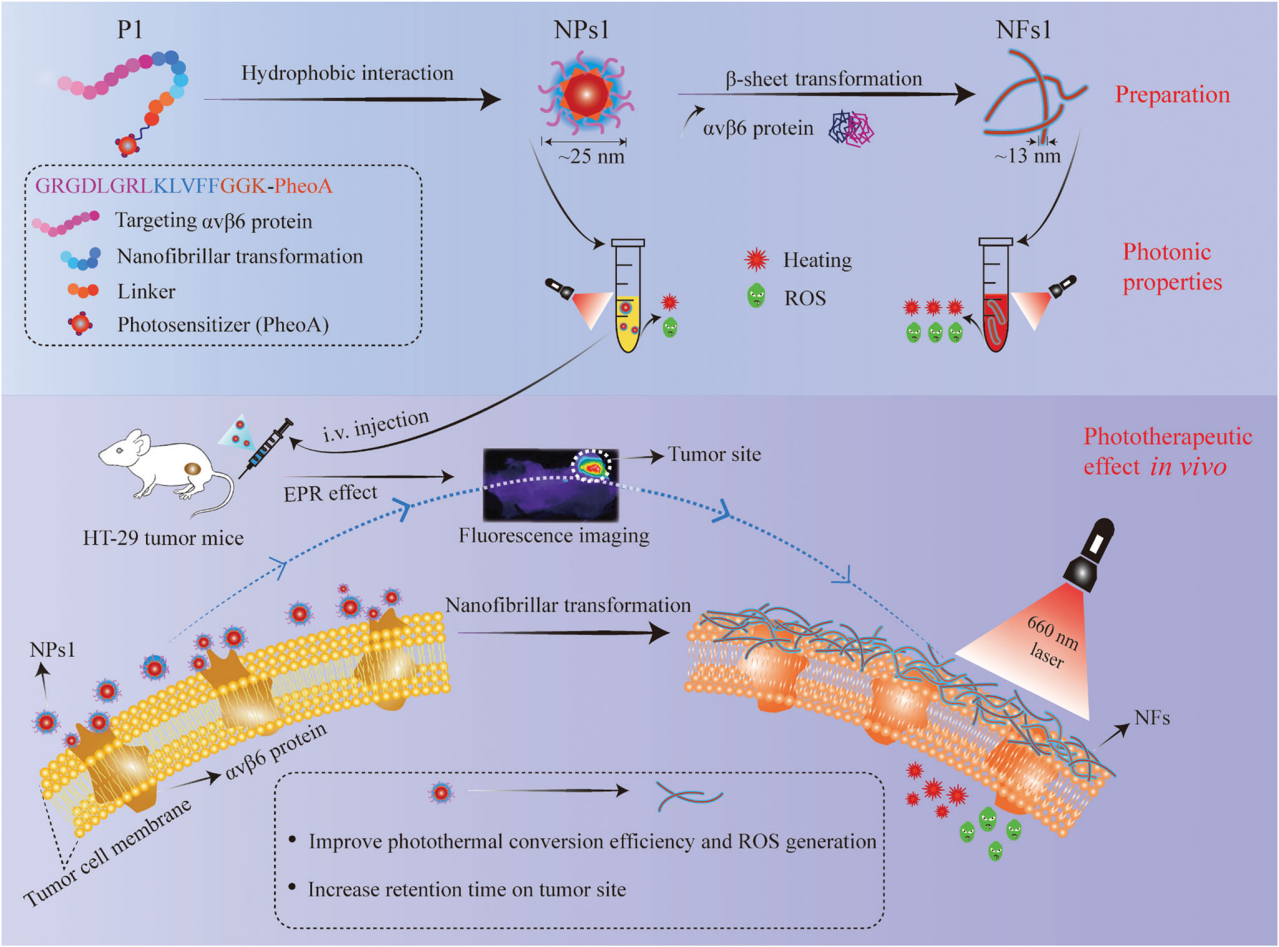
Phototherapeutic effect of smart supramolecular peptide (NPs1) on mice bearing HT-29 tumor. Schematic illustration of preparation, photonic properties, and phototherapeutic effect *in vivo* of nanofibrils.

Therefore, based on the self-assembled fibrils of KLVFF (Pham et al., 2014; Li et al., 2016; Yu et al., 2016; Zhang et al., 2020), we reported a new strategy to improve the biological properties of the photosensitizer (Scheme 1). Specifically, a smart transformable peptide monomer (GRGDLGRL-KLVFF-GGK-PheoA) was designed and synthesized. It consisted of three key parts, including (1) the hydrophilic core GRGDLGRL, which showed a high targeting ability toward the integrin αvβ6 protein on the tumor cell surface (Roesch et al., 2018), (2) the KLVFF, which can transform into nanofibrils through β-sheet (Li et al., 2016; Zhang et al., 2020), and (3) pheophorbide a (PheoA), a photosensitizer with the fluorescent ability (Xodo et al., 2012; Saide et al., 2020). Peptide GGK acted as a linker, which could be used to connect PhoeA, because lysine (K) has two amino groups, one of which could be used for dehydration condensation with the carboxyl group of PhoeA. In an aqueous condition, the peptide monomer self-assembled into nanoparticles (NPs) under the force of hydrophobic interaction. PheoA and the peptide KLVFF acted as lipophilic groups, and the peptide GRGDLGRL acted as the hydrophilic group in this process. The NPs displayed further transformation into nanofibrils (NFs) after NPs combination with the integrin αvβ6 protein on the HT-29 cancer cell surface with the help of the β-sheet-forming peptide KLVFF. We proved that the nanofibrillar aggregated state of PheoA was propitious to the pursuance of light-to-energy conversion efficiency, ROS generation, and tumor retention, which enhanced the inhibitory effect on tumors.

## 2. Materials and methods

### 2.1. Synthesis and characterization of peptide conjugates

Three peptide conjugates (P1-3) were synthesized in this study. First, GRGDLGRLKLVFFGGK(Dde)-resin, GRGDLGRLKAAGGGGK(Dde)-resin, and Fmoc-KLVFFGGK(Dde)-resin were synthesized by the standard solid-phase synthesis in sequence. The Fmoc protection group was replaced by PEG_600_. Then, PheoA containing -COOH was linked to -NH_2_ on the peptide chains after deprotection of Dde (amino protection group). Crude peptides were obtained after removing the resin. Ice ether (−20 °C) was used for preliminary purification of peptides, and preparative chromatography (QBH-P1300, Qingbohua Technology Co., Ltd., China) was used to further purify them. Finally, ESI mass spectrometry (Waters Micromass ZQ, Waters Corporation, USA) confirmed the P1-3 molecular structures.

### 2.2. Preparation and characterization of nanoparticles and nanofibrils

P1-3 were dissolved in DMSO to formulate solutions with a concentration of 4 mM. Then, the peptide solutions (5 μL) were diluted with 995 μL water to prepare the nanoparticle solutions of 1000 μL (20 μM, water content 99.5%), which were used in the following experiments. In addition, solutions (20 μM) containing different water amounts (including 0%, 20%, 40%, 60%, and 80%) were also prepared to verify the formation of NPs1 by using the ultraviolet-visible absorption (UV-Vis, Thermo Scientific NanoDrop^TM^ One, Thermo Fisher, USA) and fluorescence spectrum (excitation wavelength = 405 nm, F7000, Hitachi, Japan). The size and zeta potential of the NPs were measured with dynamic light scattering (DLS, Zetasizer Nano S90, Malvern Company, UK). A transmission electron microscope (TEM, JEM-1400 Flash, JEOL Corporation, Japan) was used to observe the morphology of NPs. To prepare the nanofibrils (NFs), nanoparticles (20 μM) were cultured with the integrin αvβ6 protein (recombinant human integrin αvβ6/ITGAV&ITGB6 heterodimer protein, ACROBiosystems) for 24 h at the molar ratio of peptide/protein 500:1 (the same as the following experiment). Fluorescence spectrum, circular dichroism (CD, Chirascan^TM^ V100, Applied Photophysics Ltd., UK), Malvern Zetasizer, and TEM confirmed the morphological transformation of nanoparticles to nanofibrils. In addition, the NPs1 was also incubated with 10% (*v/v*) fetal bovine serum (FBS) for 24 h to verify that the proteins in the serum did not interact with NPs1 through DLS and TEM.

### 2.3. Cell culture

The human colorectal cancer cell line HT-29 and breast cancer cell line MDA-MB-231 were purchased from BeNa Culture Collection Biotechnology Co., Ltd. (Beijing, China). The HepG2 cell line was obtained from Guangzhou Jenniobio Biotechnology Co., Ltd. (Guangzhou, China). These cell lines were cultured in complete Dulbecco’s modified eagle medium (DMEM) containing 10% (*v/v*) FBS, 100 U/mL penicillin, and 100 ug/mL streptomycin, and were incubated at 37 °C with 5% CO_2_.

### 2.4. Transformation of NPs1 into NFs on cancer cell surface

Cells were seeded in glass-bottom dishes at a density of 2 × 10^5^/well for 12 h. To validate the interaction of NPs1 with the αvβ6 protein, HT-29 cancer cells were treated with NPs1-3 (20 μM) for 8 h, and then were fixed with 2.5% glutaraldehyde for 30 min. Then, the anti-αvβ6 antibody (1:100) was cultured with cells for 30 min, followed by Green Alexa Fluor 488-labeled Goat Anti-Rabbit IgG(H + L) (1:100, excitation wavelength = 488 nm) and incubated for 30 min. After being washed with PBS three times, confocal laser scanning microscopy (CLSM, SP8, Leica, Germany) was used to observe the fluorescence distribution of different NPs (excitation wavelength = 405 nm, the same as following CLSM experiments) and αvβ6 protein on HT-29 cells. We used the Pearson correlation coefficients to quantify the degree of colocalization between two fluorescence (NPs and αvβ6 protein) according to the previous study (Adler & Parmryd, 2010). Besides, the distribution of NPs1 in αvβ6-negative cancer cells MDA-MB-231 and HepG2 was also observed after incubation with NPs1 (20 μM) for 8 h, respectively. HT-29 cancer cells were treated with NPs1 (20 μM) for 1, 8, and 24 h prior to CLSM imaging to investigate the accumulation of NPs1 within 24 h. A scanning electron microscope (SEM, S4800, Hitachi, Japan) was used to further validate the transformation of NPs1 into NFs on the cell surface. NPs1–3 (20 μM) were incubated with cells on the cover glass for 24 h, and 2.5% glutaraldehyde was used to fix the cells for 30 min. Cell samples were coated with gold for SEM imaging after drying.

### 2.5. Stability of NFs structures

For the stability of NFs, HT-29 cells were incubated with NPs1 (20 μM) for 8 h; then, the complete DMEM was removed and replaced with flesh complete DMEM without NPs1 for another 16 h and 40 h. Next, 2.5% glutaraldehyde was used to fix the cells for 30 min before CLSM imaging. The cells with NPs2 and NPs3 were treated the same way. For the cellular uptake of NPs2 and NPs3, HT-29 cells were incubated with NPs2 and NPs3 for 8 h respectively; then, the complete DMEM was removed and replaced with flesh complete DMEM without NPs2 and NPs3 for another 8, 16, and 40 h. After that, a green lysosome tracker was added to the complete DMEM (50 nM), and incubated with the cells for 30 min. Using CLSM, we observed the intracellular distribution of NPs2 and NPs3.

### 2.6. Detection of photothermal conversion efficacy and ROS generation in solution

A 660 nm laser (MW-SGX-660, Changchun Laser Optoelectronics Technology Co., Ltd., China) was used in the following experiment. To study the effect of concentration on photothermal activity, the temperature of various concentrations of NPs1 and NFs1 solutions (1, 10, 20, 50 μM) was measured via a thermal imaging camera (C3-X, FLIR Systems, USA) after laser irradiation for 120 s (0.4 W/cm^2^). To compare the photothermal effect of NPs1 and NFs1, irradiation-cooling cycles (laser for 120 s and then cooling for 90 s) were repeated four times, recording the temperature change every 15 s. For photodynamic analysis, a 2,7-Dichlorodihydrofluorescein diacetate (DCFH-DA) fluorescent probe was used to detect the ROS generation, according to a previous study. In brief, DCFH-DA was hydrolyzed by NaOH (0.01 N) to DCFH (2′,7′-Dichlorodihydrofluorescein) and neutralized with PBS before use. By adding DCFH solution, different NPs (20 μM) with and without αvβ6 protein was exposed to the laser (0.4 W/cm^2^, 30 s), and the ROS generation was recorded by a fluorescence microplate reader (excitation wavelength = 488 nm, SpectraMax i3x, Molecular Devices, USA).

### 2.7. Cytotoxicity evaluation

An MTT assay was used to evaluate cell viability. In brief, different concentrations of NPs (1, 5, 10, 20, 35, 50 μM) were prepared by DMEM and added to each well after culturing cells at a density of 8 × 10^3^/well (*n* = 3). After incubation for 8 h, the medium was removed and replaced with flesh complete DMEM. Then, the cells were exposed to light irradiation for 30 s (660 nm, 0.4 W/cm^2^), and further incubated for 16 h. After that, a freshly prepared MTT solution (1 mg/mL, 0.1 mL) was added to each well. After incubation for 4 h, the MTT medium was removed, and DMSO (200 μL) was added to each well immediately prior to detection of the optical density (OD_490_) using a microplate reader (BioTek Instruments Inc., Winooski, USA). The cell viability was calculated by the following equation: cell viability (%) = (OD treatment/OD control) × 100%. To study the respective contribution of PTT and PDT to phototherapy, cancer cells were treated with various concentrations of NPs1 for 8 h. N-acetylcysteine (NAC) was added to the medium 2 h before laser irradiation, which could suppress the PDT effect by quenching ROS. To assess the PTT effect, cell culture plates were put into 0 °C ice water during the irradiation time. In addition, to validate the retention effect of NFs, cells were cultured with complete DMEM containing different concentrations of NPs for 8 h. The medium was removed, and the cells were cultured with flesh medium for 16 h, and then exposed to light irradiation for 30 s (660 nm, 0.4 W/cm^2^). After that, complete DMEM incubation for 16 h was necessary for the cancer cells before the MTT assay.

### 2.8. Apoptosis evaluation and ROS generation

Cancer cells were seeded in 12-well plates at a density of 5 × 10^5^/well, and different NPs (20 μM) were added to each well. After incubation for 8 h, they were washed and incubated with flesh complete DMEM. Then, the cells were exposed to light irradiation for 30 s (660 nm, 0.4 W/cm^2^) and were further incubated for 16 h. By adding the Annexin V-FITC/PI Apoptosis Detection Kit for 20 min, apoptosis of the cancer cells was observed under the inversion microscope (ECLIPSE Ts2R, Nikon Corporation, Japan) and evaluated via flow cytometry (BD FACSCanto II, Becton, Dickinson and Company, USA). The wavelength excitations of FITC and PI were 488 nm and 535 nm, respectively. To validate the retention of NFs, after being cultured with different NPs for 8 h, cells were washed, and further incubated with flesh complete DMEM for 16 h followed by laser irradiation (30 s, 0.4 W/cm^2^). Further incubation for 16 h was needed prior to the addition of the Annexin V-FITC/PI Apoptosis Detection Kit and detection. The ROS generation was determined in the same way. However, instead of the Annexin V-FITC/PI Apoptosis Detection Kit, the DCFH-DA Cellular Reactive Oxygen Assay Kit (excitation wavelength = 488 nm) was used to treat cells for 30 min before laser irradiation, and then the intracellular ROS was detected via flow cytometry.

### 2.9. Transformation of NPs1 into NFs in vivo

All the animal experiments were performed in accordance with the guidelines approved by the Institutional Animal Care and Use of Committee of Beijing University of Chinese Medicine (BUCM-4-2022021802-1016). Male Balb/c nude mice were injected with HT-29 cells (5 × 10^6^) to establish a subcutaneous colorectal tumor transplantation model. The volume of tumors was measured by the following formula: *V* = 0.5 × L × W^2^. When the tumor grew to 100 mm^3^, the mice were divided into three groups (three mice in each group) and intravenously injected with different NPs PBS solutions (7.5 mg/kg). Three days after administration, the tumor tissues were fixed with 2.5% glutaraldehyde and stained with DAPI (excitation wavelength = 364 nm) for CLSM observation. In addition, the tumor tissues were also made into TEM samples to validate the nanofibrils.

### 2.10. Fluorescence distribution and photothermal imaging in vivo

For fluorescence distribution imaging, three groups of tumor mice (three mice in each group) were injected with different NPs PBS solutions (7.5 mg/kg) and then were imaged by a multifunctional *in vivo* imager (excitation wavelength = 405 nm, MIIS, Molecular Devices, USA) at different time points including 24, 72, 120, and 168 h. Tumor tissues and organs (including heart, liver, spleen, lung, kidney, intestine, muscle, and skin) were collected after 168 h to image. For photothermal imaging *in vivo*, the tumor regions were irradiated for 120 s (0.4 W/cm^2^) after injection with different NPs PBS solutions (7.5 mg/kg) for 24, 72, 120, and 168 h, respectively, and the temperature was recorded by a Flir camera.

### 2.11. Phototherapeutic study in vivo

Tumor mice were divided into five groups (6 mice in each group) and then treated with different solutions (7.5 mg/kg): PBS, NPs1, NPs1 (laser), NPs2 (laser), and NPs3 (laser). After administration for 24, 48, 72, 96, 120, and 144 h, the 660 nm laser was used to irradiate the tumor regions for 120 s (0.4 W/cm^2^). Mice were treated once per week for two cycles. The weight of the mice and the volume of the tumor was measured every two days for a total of two weeks. Then the tumor tissues were collected and weighted. In addition, tumor tissues and important organs (including heart, liver, spleen, lung, kidney, intestine, muscle, and skin) were further processed by hematoxylin and eosin (H&E) staining.

### 2.12. Statistical analysis

Data are presented as mean ± s.d. Student’s *t*-test (two-tailed) was used for intergroup analysis, and one-way analysis of variance (ANOVA) was used for multiple group analysis. A *p* value < .05 was considered statistically significant.

## 3. Results and discussion

### 3.1. Synthesis, preparation, and characterization of nanoparticles and nanofibrils

**Figure 1.**
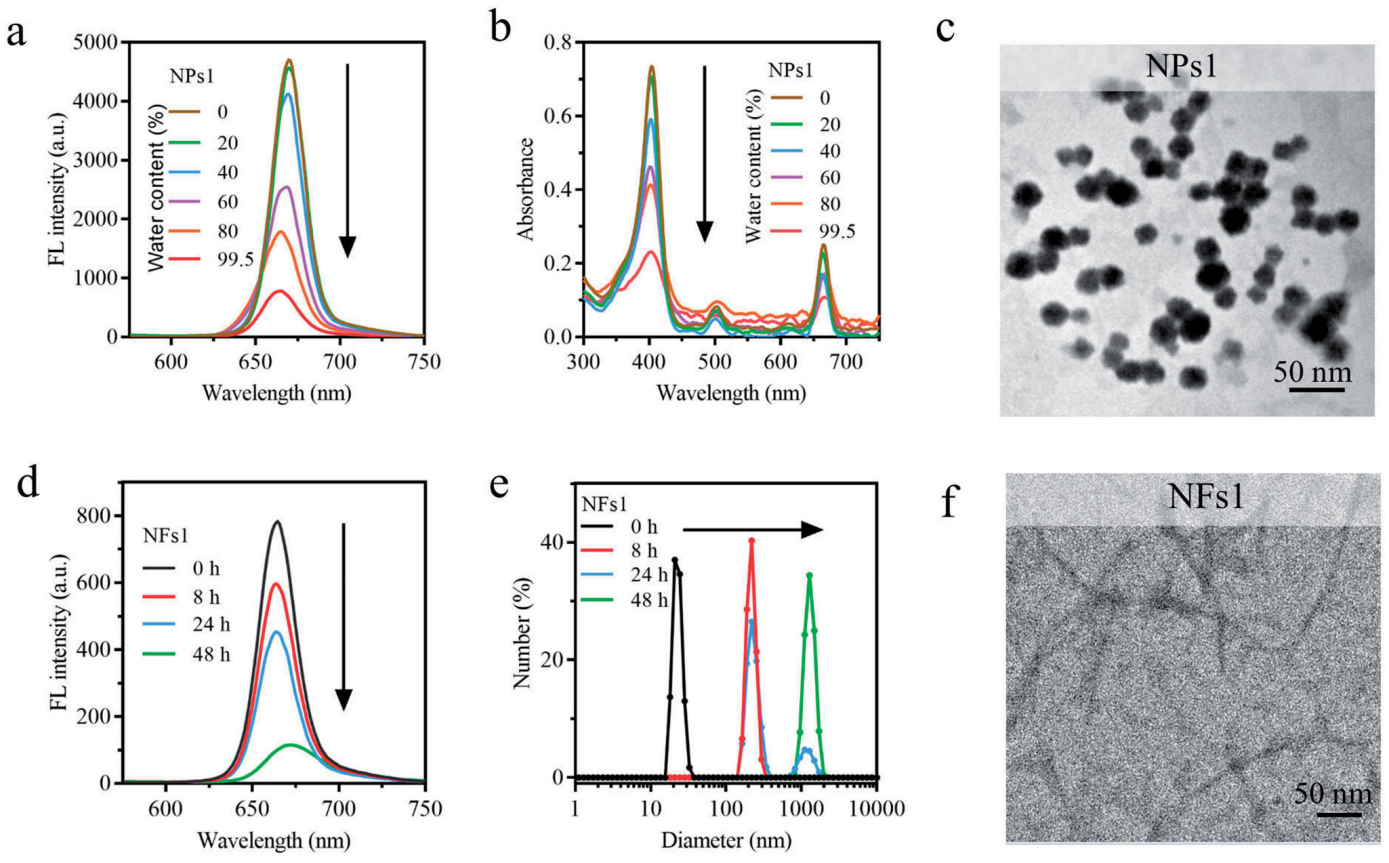
Self-assembly, and nano fibrillar transformation of smart peptide monomer GRGDLGRL-KLVFF-GGK-PheoA (P1). Fluorescent intensity (a) and absorbance (b) of NPs1 solutions with different water content (including 0%, 20%, 40%, 60%, 80%, 99.5%). The excitation wavelength was 405 nm. (c) TEM images of fresh NPs1 at the water and DMSO ratio of 995:5. Fluorescent intensity (d) and variation in size distribution (e) of NFs1 (NPs1 incubated with integrin αvβ6 protein at the molar ratio of 500:1) within 48 h. (f) TEM image of NFs1 transformed by initial NPs1 after incubation with αvβ6 protein for 24 h. The concentration of NPs1 related to these experiments was 20 µM, and all these experiments were repeated three times.

The peptide of GRGDLGRL-KLVFF-GGK-PheoA (P1) was synthesized by standard solid-phase synthesis, and characterized by ESI mass spectrometry. Compared with P1, negative control peptides P2 (GRGDLGRL-KAAGG-GGK-PheoA) lacking KLVFF and P3 (PEG_600_-KLVFF-GGK-PheoA) lacking GRGDLGRL were also synthesized (Supplementary Figure S1). The fluorescence and UV-Vis spectrum were used to verify the process of self-assembly. With the water content increase in the mixed solvent, both the absorption peak and fluorescence peak intensity of the P1 solutions gradually decreased with the changes in the aggregate state of PheoA (Figure 1(a,b)) (Xodo et al., 2012), indicating that the nanoparticles of P1 (NPs1) were gradually formulated. The morphologies and diameters of NPs1–3 were detected through transmission electron microscopy (TEM) which were found to be around 25 nm (Figure 1(c), Supplementary Figure S2(a)), and remained stable for seven days in a phosphate buffer solution (PBS), according to the DLS results (Supplementary Figure S2(b)). The zeta potential studies showed that NPs1–3 had a positive charge at 28.1, 24.2, and 27.4 mV, respectively (Supplementary Figure S2(c)).

To confirm the structural transformation of NPs1, the soluble integrin αvβ6 protein was incubated with different NPs. As the study showed, the fluorescent intensity of NPs1 was reduced by around 80% after incubation with αvβ6 protein for 48 h due to the PheoA property of aggregation-caused quenching (ACQ) (Figure 1(d)), which indicated that the structure of NPs1 underwent dramatic reconstitution. The suppression of fluorescence was conducive to the generation of more heat and ROS (Abbas et al., 2017; Sun et al., 2020). According to the DLS research, the diameter of NPs1 gradually increased from about 25 nm to 1281 nm after incubation with αvβ6 protein for 48 h (Figure 1(e)), and no obvious changes in the negative control groups’ (NPs2 and NPs3) diameters were observed after incubation (Supplementary Figure S3(a)). Besides, the NPs1 treated with 10% FBS had no change in diameter, indicating that NPs1 was stable and had no interaction with other proteins in serum (Supplementary Figure S3(a)). From the result of the TEM, slenderer nanofibrils (NFs1) with a width diameter of around 13 nm were found rather than the initial irregular spherical structure of NPs1 at around 25 nm after incubation with αvβ6 protein (Figure 1(f)). NPs1 treated with 10% FBS and negative control groups treated with αvβ6 protein retained an irregular spherical structure (Supplementary Figure S3(b)). Further study including the zeta potential and circular dichroism also validated the transformation of NPs1 into NFs1. Over time during the transformation, the charge of NPs1 became converse from 28.1 to −8.4 mV, and the negative control groups retained a positive charge at around 24 mV (Supplementary Figure S2(c)). CD spectroscopy could show the characteristic peaks for the β-sheet structure of peptides (Li et al., 2016; Zhang et al., 2020). A positive signal at 194 nm and a negative signal at 216 nm in the NFs1 CD spectrum could be due to the β-sheet-forming peptide KLVFF, which mediated the transformation of nanofibrils (Supplementary Figure S4). Although NPs3 also contained the KLVFF peptide, it showed no significant structural transformation in the presence of the integrin αvβ6 protein for 24 h. These results showed that two essential domains including the ligand peptide GRGDLGRL and the fibril-forming peptide KLVFF may be the cause of the structural transformation through the ligand-receptor interaction and β-sheet.

### 3.2. Transformation and stability of NFs structures

**Figure 2.**
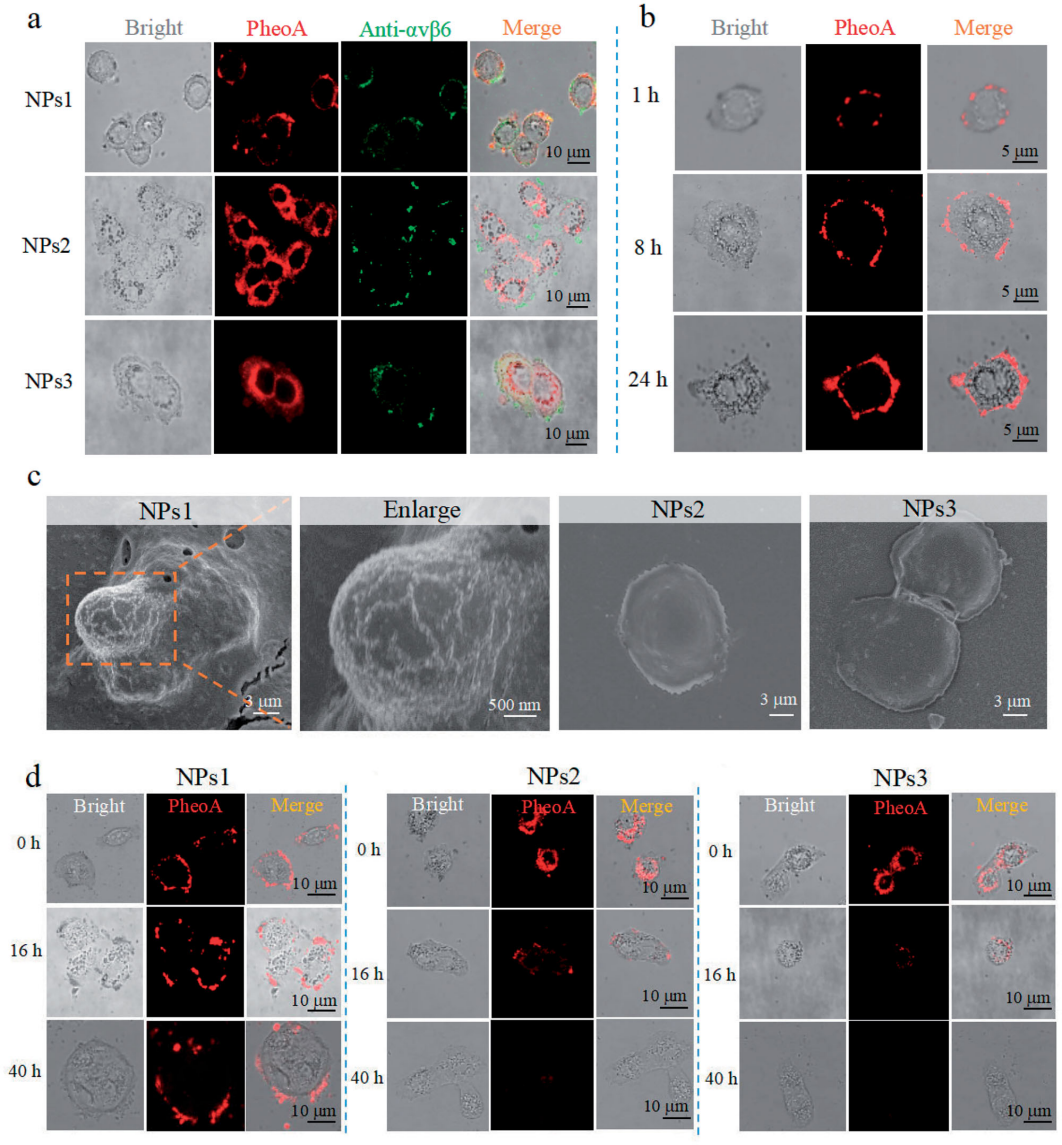
Transformation and retention of NPs on the cancer cells' surface. (a) Fluorescence distribution images of integrin αvβ6 protein and different NPs (incubation with cells for 8 h) on HT-29 tumor cells. Integrin αvβ6 protein: Green color (excitation wavelength = 488 nm). PheoA: Red color (excitation wavelength = 405 nm). (b) Fluorescence distribution images of cancer cells treated with NPs1 for 1, 8, and 24 h. (c) SEM images of tumor cells incubation with different NPs for 24 h. (d) Fluorescence signal retention on tumor cells. Cells were incubated with different NPs for 8 h, respectively, then the complete DMEM was removed and replaced with flesh complete DMEM without NPs for another 16 and 40 h. The concentration of NPs related to these experiments was 20 µM, and all these experiments were repeated three times.

Integrin αvβ6 is considered to be a prognostic indicator for its poor expression or absence in healthy adults but upregulation in several kinds of cancers (Niu & Li, 2017). It is also involved in numerous malignant behaviors of tumor progression and relates to the survival time of patients (Zhang et al., 2008; Bandyopadhyay & Raghavan, 2009). Therefore, integrin αvβ6 has been considered for tumor imaging, and as a tumor therapy target. The anti-αvβ6 antibody was used to detect αvβ6 protein on HT-29 colorectal cancer cells' surface imaged by a fluorescent green secondary antibody (Liang et al., 2015; Slack et al., 2016). After incubation with NPs1 for 8 h, the cell periphery was wrapped by red fluorescence (PheoA), which almost overlapped with the green light (αvβ6 protein) (Figure 2(a)). As the Supplementary Figure S5 showed, the Pearson correlation coefficient for NPs1 and αvβ6 protein colocalization in the cell surface was +0.85, indicating that NPs1 had a specific recognition of the αvβ6 protein on HT-29 cells (+1, perfect correlation; −1, perfect but negative correlation; 0, the absence of a relationship) (Bello et al., 2016). In addition, its fluorescent intensity gradually increased in time-dependence, meaning the continuous formation and accumulation of nanofibrils (Figure 2(b)). As for the negative control groups NPs2 and NPs3, red fluorescence appeared in the cell cytoplasm rather than staying at the cell surface (Figure 2(a)), and their Pearson correlation coefficients were +0.18 and +0.17, respectively, indicating that NPs2 or NPs3 and αvβ6 protein had no colocalization (Supplementary Figure S5). MDA-MB-231 and HepG2 cells which rarely expressed αvβ6 protein were also treated with NPs1 for 8 h, respectively (Vellon et al., 2005; Patsenker et al., 2010). As expected, NPs1 did not stay on these cell surfaces in the absence of αvβ6 protein (Supplementary Figure S6). The results of SEM further revealed the presence of a nanofibrillar network on the HT-29 cell surface. After incubation with NPs1, the nanofibrillar network was gradually formed over 24 h. On contrary, no obvious nanofibrillar structure was found on the cell surface of the negative control groups (Figure 2(c)). These proved that the structural changes triggered by αvβ6 protein, and induced by the fribril-forming peptide KLVFF still worked on living cells. To study the stability of nanofibrils, different NPs were used to treat the cells for 8 h; then, they were removed for another 16 h and 40 h. Despite the lack of NPs1, the red fluorescence in the cell periphery remained at a stable intensity. This meant that the nanofibrils could be stably presented at the cell surface by reducing the enzyme solution, which is significant for PheoA to improve the tumor retention ability (Figure 2(d)). The cells were treated in the same way with NPs2 and NPs3. The red fluorescence gradually decreased, and almost disappeared within 40 h (Figure 2(d)). Cellular uptake studies of NPs2 and NPs3 revealed that most of them were degraded in lysosomes (Supplementary Figure S7). In a word, the fluorescence distribution assay on cancer cells verified the transformation of NPs1 into stable nanofibrils via the ligand-receptor interaction and β-sheet.

### 3.3. Transformation and stability of NFs structures

**Figure 3.**
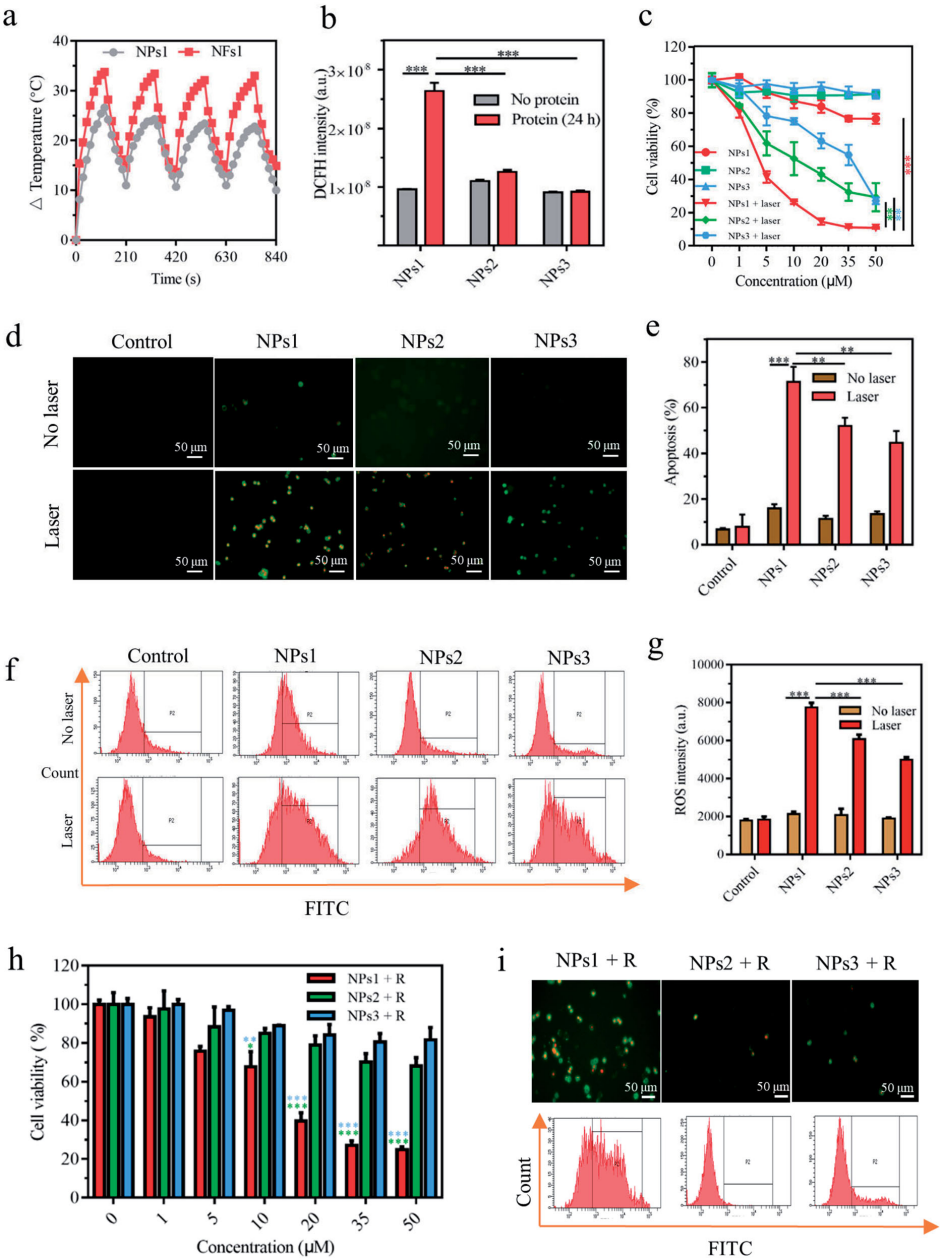
The therapeutic effect of different NPs *in vitro*. (a) Temperature variation of NPs1 and NFs1 (NPs1 incubation with αvβ6 protein for 24 h) during four irradiation-cooling cycles (laser irradiation for 120 s, and then cooling for 90 s). The concentration of NPs1 and NFs1 was 50 μM. (b) ROS generation of different NPs (20 μM) with/without αvβ6 protein by using DCFH fluorescence probe after irradiation for 30 s (excitation wavelength = 488 nm). (c) Cell viability of HT-29 cancer cells after incubation with different NPs for 8 h, with irradiation (30 s) or not. The fluorescence images (Green color: apoptosis cells; Red color: necrosis cells) (d) and quantitative analysis (e) of apoptosis cells after treatment with different NPs (20 μM) for 8 h, then irradiation for 30 s or not. The excitation wavelengths of FITC and PI were 488 nm and 535 nm, respectively. Cellular ROS generation via flow cytometry (f) and relative quantitative analysis (g). DCFH-DA Cellular Reactive Oxygen Assay Kit was used to detect ROS after treating cancer cells with different NPs for 8 h with/without irradiation for 30 s (excitation wavelength = 488 nm). (h) Cell viability of long-term retention of NPs. Treating cancer cells with different NPs for 8 h, and replaced with a new flesh medium for 16 h prior to irradiation for 30 s, then the viability of the cells were assayed by MTT. (i) Apoptosis and ROS generation of long-term retention of NPs. Before irradiation for 30 s, cancer cells were treated with different NPs for 8 h plus another 16 h with fresh medium. Using an inversion microscope to detect apoptosis, and flow cytometry to detect ROS generation. The irradiation powder density remained at 0.4 W/cm^2^ in these experiments. Data were presented as mean ± s.d. (*n* = 3, *P < .1, **P < .01, ***P < .001).

Depending on the mechanism of action, phototherapy can be divided into PDT and PTT (Abbas et al., 2017). In an oxygen-dependent manner, PDT can induce cell death through the mechanism of apoptosis or necrosis by activating the photosensitizer to generate ROS (Xodo et al., 2012). As for PTT, the therapeutic effect connects to the photothermal conversion ability because of the susceptibility of cancer cells to heat (Han & Choi, 2021). To study the effects of the nanofibrils for phototherapy, NPs1 was incubated with αvβ6 protein for 24 h; then, it was subjected to continuous irradiation for 120 s with a power density of 0.4 W/cm^2^. The temperature of the NFs1 solution (50 μM) increased by 34.0 °C during the first laser irradiation, while the temperature of the NPs1 solution only increased by 26 °C. The photothermal conversion efficiency of NFS1 reached 54%, which was higher than that of NPs1 (41%) by calculation (Ren et al., 2015; Zhang et al., 2017). In addition, the temperature of the NFs1 solution remained stable during the irradiation-cooling cycles four times, while the NPs1 showed a decline in photothermal performance, revealing the superior photostability and thermal stability of NFs1 (Figure 3(a), Supplementary Figure S8(a)). There were no changes to the photothermal conversion efficacy of the negative control groups after incubation with αvβ6 protein (Supplementary Figure S8(b,c)). The PDT study showed the same trend as PTT. The ROS generation of NFs1 detected by the fluorescent probe DCFH-DA was more than twice that of NPs1 (Figure 3(b)). This might be due to the fact that the inhibition of fluorescence is conducive to pursuing a high phototherapeutic effect. When the fluorescence of PheoA was quenched for the aggregated state, the excited electron had to release energy through nonradiative relaxation to produce heat and intersystem crossing to generate ROS (Zhao et al., 2020). In addition, the stable nanofibrils probably increased the photothermal stability of the PheoA, which was beneficial for the therapeutic effect.

Our study confirmed that the promotional phototherapeutic effects of nanofibrils still had effects on cultured cells. After being treated with different NPs for 8 h, and then laser irradiation or not for 30 s, the cell viability was measured by MTT assay. These results showed the slightly suppressed effects of NPs1–3 in the absence of irradiation. After irradiation, the NPs1 inhibited the proliferation of cancer cells in a dose-dependent manner, and the cell viability was found to be around 10% at a concentration of 50 µM, which was better than the negative control groups NPs2 and NPs3 with cell viability at 29% and 27%, respectively (Figure 3(c)). Further studies were performed to detect the apoptotic and necrotic cells by using the Annexin V-FITC/PI Apoptosis Detection Kit. The cells treated with NPs1 exhibited more bright green fluorescence (apoptosis) and red fluorescence (necrosis) after irradiation for 30 s than the negative control groups under an inversion microscope (Figure 3(d)). According to the flow cytometry study, the rate of apoptotic and necrotic cells induced by NPs1 increased to 71% compared with the control groups whose rates were 52% and 45%, respectively (Figure 3(e)). This result was consistent with the generation of ROS detected by the DCFH-DA fluorescent probe. Green fluorescence was found in the majority of cells treated with NPs1, and the fluorescent intensity at around 8000 was higher than that of NPs2 and NPs3 at around 6000 and 5000, respectively (Figure 3(f,g)). Taken together, treatment with NPs showed slight inhibition for cells, but after irradiation, the difference in the inhibitory effect between NPs1 and the negative control groups emerged. The more potent cell killing effects of NPs1 could be associated with the superior efficient photothermal conversion and increasing generation of ROS. In addition, the respective contribution of PTT and PDT for the phototherapy was also revealed by using N-acetylcysteine (NAC) to quench ROS, and cold (0 °C) to avoid the photothermal effect. As Supplementary Figure S9 shows, the inhibition rate of the NPs1 PTT effect was found to be 54% after irradiation, which is over twice higher than that of the PDT effects with a 20% cell kill at 50 µM.

Further study revealed the effect of the long-term retention of the nanofibrillar network on HT-29 cancer cells. After incubation with different NPs, the medium was replaced with flesh complete DMEM for another 16 h. As expected, the NPs1 still exhibited significant inhibition with the 75% cell kill at 50 µM, while the inhibitory effects of the negative control groups reduced sharply (Figure 3(h)). Moreover, the prolonged retention of the nanofibrillar network was also evaluated by detecting apoptosis and ROS. Compared with the negative control groups, NPs1 could induce cell apoptosis and necrosis, and generate ROS at a high level (Figure 3(i)). These results presented the synchronous change as the fluorescence on the cell surface. In brief, the stable nanofibrillar network ensured long-term inhibitory capacity, and the endocytosed negative control groups NPs2 and NPs3 were gradually hydrolyzed by the enzyme, which showed less damage for cells.

### 3.4. In vivo evaluation of fibrillary-transformable nanoparticles

**Figure 4.**
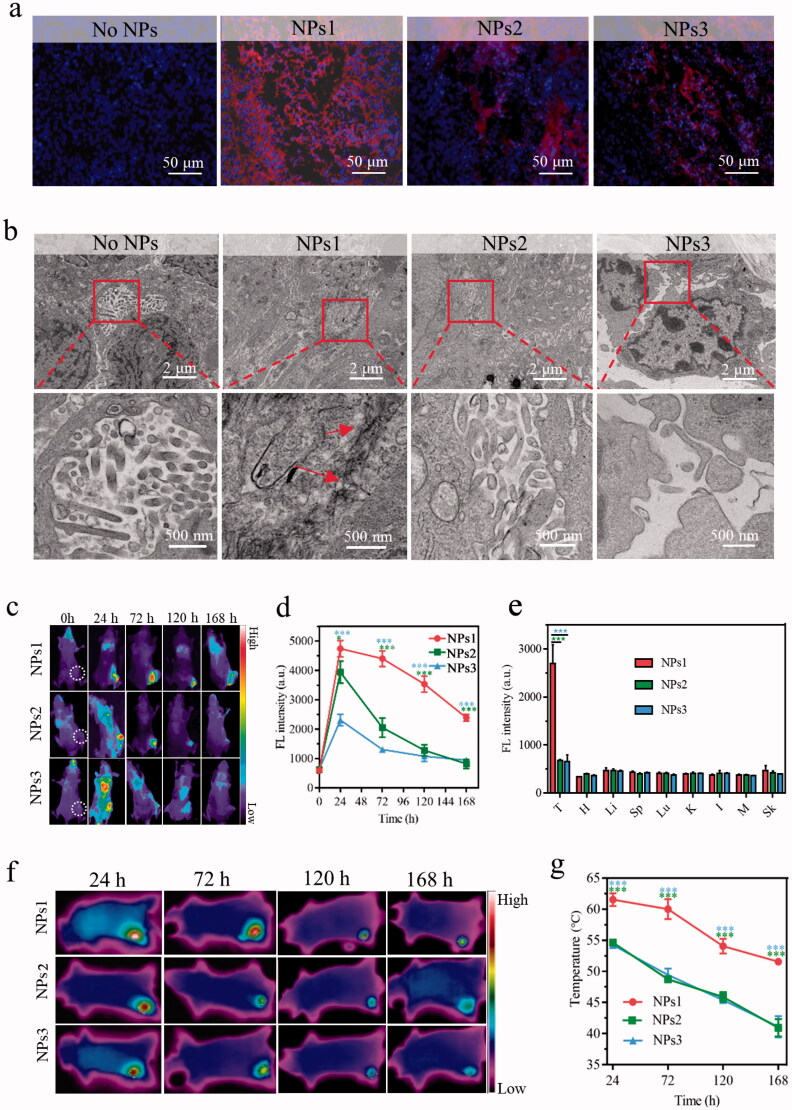
Distribution, transformation, and photothermal research *in vivo*. (a) The fluorescence signal of tumor tissue after i.v. injection with different NPs for 72 h (Blue color: DAPI, excitation wavelength = 364 nm; Red color: PheoA, excitation wavelength = 405 nm). (b) TEM images of nanofibrillar transformation in tumor tissue after treatment with NPs for 72 h. Part of fluorescence distribution images (c) and quantitative analysis (d) of HT-29 tumor-bearing mice at different time points including 24, 72, 120, and 168 h, after treatment with different NPs (*n* = 3, excitation wavelength = 405 nm). (e) Excised tumors and several organs quantitative analysis after i.v. injection with different NPs for 168 h (T, H, Li, Sp, Lu, K, I, M, and Sk represented tumor, heart, liver, spleen, lung, kidney, intestine, muscle, and skin, respectively). IR thermal images (f) and temperature variation (g) in tumor tissue after injection with different NPs at 24, 72, 120, and 168 h with irradiation for 120 s (*n* = 3). The dose of NPs in these experiments was 7.5 mg/kg, and the data were presented as mean ± s.d. (*P < .1, **P < .01, ***P < .001).

To study the fibrillary-transformable ability of NPs1 *in vivo*, HT-29 colorectal cancer-bearing mice were injected with different NPs PBS solutions in the tail vein for 72 h. Tumor tissue was stained with DAPI (blue) and observed under CLSM. The results showed that the strong red fluorescence (PheoA) was distributed throughout the tumor tissue after treatment with NPs1. In the negative control groups, they were only distributed in some areas of the tumor, and their fluorescent intensity was weaker than that of NPs1 (Figure 4(a)). This might be due to the transformation of NPs1 into nanofibrils, which caused long-term retention in the tumor region, and the TEM studies finally confirmed the speculation. An abundance of nanofibrils was observed in the tumor tissue, while the negative control groups had no such nanostructure (Figure 4(b)). Biodistribution studies revealed the difference in NPs *in vivo* stability. Three groups of tumor mice were injected with NPs PBS solutions; then, they were imaged by an animal imager at different time points including 24, 72, 120, and 168 h, respectively. All of the NPs were mainly distributed in the tumor region and rarely absorbed by the organs (Figure 4(c), Supplementary Figure S10). As expected, the fluorescence of NPs1 declined more slowly than NPs2 and NPs3 within 168 h after injection. Compared with the fluorescence of negative control groups, which almost disappeared (fluorescence intensity at around 700), nearly 50% signal remained for NPs1 at 168 h (Figure 4(d)). Consistently, the excised tumor tissue treated with NPs1 showed stronger fluorescence than that of NPs2 and NPs3 (Figure 4(e), Supplementary Figure S11). These results could be due to the in situ fibrillary transformation in the tumor, which enhanced the retention of NPs1. The photothermal imaging study on tumor mice was investigated to validate the superior photothermal conversion of nanofibrils. After treatment with NPs by intravenous (i.v.) injection, the tumor site was irradiated for 120 s (0.4 W/cm^2^) at different time points including 24, 72, 120, and 168 h, and the infrared camera was used to detect the temperature. Consistent with the fluorescent changes of the distribution study, the temperature in the tumor region reached its peak at 24 h, then gradually declined over the next few days, and the negative control groups declined more quickly than NPs1 (Figure 4(f)). In particular, the temperature of the groups treated with NPs1 reached 62 °C at 24 h, whereas only 55 °C and 54 °C were reached by NPs2 and NPs3, respectively. This could be attributed to the formation of nanofibrils. As expected, the tumor temperature maintained a high level of 52 °C after 168 h. For the negative control groups NPs2 and NPs3, the tumor temperature was detected to be around 41 °C, which was insufficient to induce irreversible damage (Figure 4(g)) (Jaque et al., 2014). In summary, with the help of superior photothermal conversion efficiency and prolonged retention time on the tumor, we can achieve the goal of one dose of lasting irradiation.

**Figure 5.**
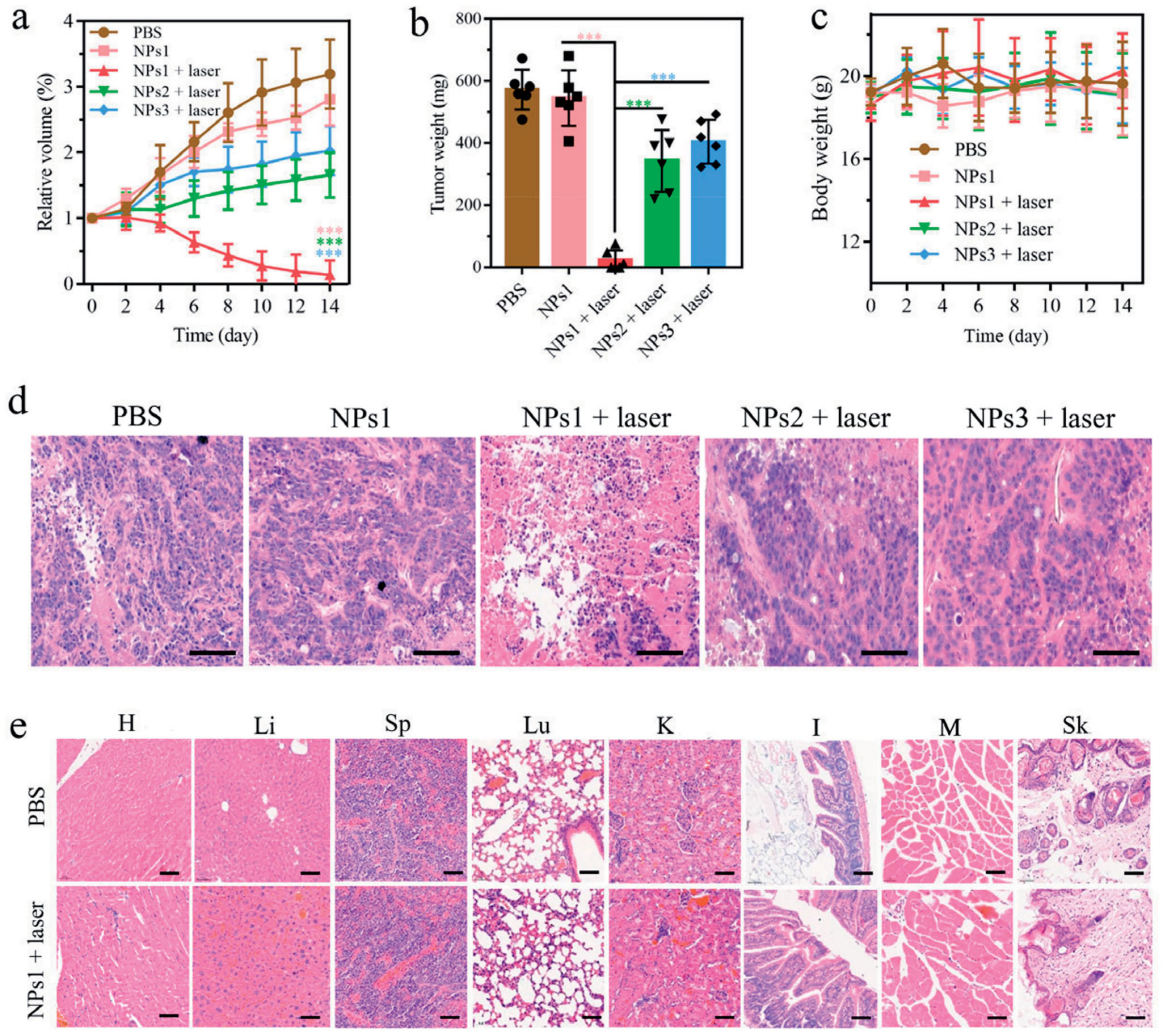
*In vivo* antitumor activity of different NPs. (a) Changes in tumor volume of five groups including PBS, NPs1, NPs1 (laser), NPs2 (laser), NPs3 (laser), during 14 days treatment (*n* = 6). (b) The final tumor weight of five groups after 14 days of treatment. (c) Variation of the body weight within 14 days of treatment. (d) H&E staining of excised tumor tissue after the phototherapeutic treatment, the scale bar was 50 μm. (e) H&E staining of important organs in the groups of PBS and NPs1 (laser), the scale bar was 50 μm (T, H, Li, Sp, Lu, K, I, M, and Sk represented tumor, heart, liver, spleen, lung, kidney, intestine, muscle, and skin, respectively). The dose of NPs in these experiments was 7.5 mg/kg, and the data were presented as mean ± s.d. (*P < .1, **P < .01, ***P < .001).

Mice bearing HT-29 colorectal cancer were used to confirm the therapeutic efficacy of NPs1 by dividing them into five groups: PBS, NPs1, NPs1 (laser), NPs2 (laser), and NPs3 (laser). As Figure 5(a,b) shows, tumors treated with NPs1 (laser) almost disappeared after 660 nm laser irradiation for two weeks, while the NPs1 group without irradiation had no tumor suppression. In the other two groups treated with NPs2 (laser) and NPs3 (laser), the tumor volume gradually increased during the therapeutic progress, and the antitumor abilities were much lower than that of NPs1 (laser). During the study, the body weight of the administration groups showed no loss, which indicated that the nanomaterials were biocompatible (Figure 5(c)). Additionally, the H&E staining study showed that the group of NPs1 (laser) caused massive tumor tissue necrosis as well as a shrinking or disappearing nucleus and ruptured cytoplasm. As for the other four groups, most of the nucleus of tumor tissue remained intact (Figure 5(d)). H&E staining of different organs in the NPs1 (laser) and PBS groups revealed that there was no difference in their histopathology, which probably confirmed the nontoxicity of the nanomaterials (Figure 5(e)). In brief, these observations demonstrated the superior phototherapeutic efficacy of NPs1 in inhibiting tumor growth but almost no secondary action for important organs.

## 4. Conclusions

Herein, we reported an in situ smart peptide nanomaterial to improve the bioavailability of PheoA. The peptide monomer underwent structural changes twice, from a long chain to nanoparticles in aqueous condition, and then a gradual transformation into supramolecular nanofibrils induced by receptor (integrin αvβ6) and the fribril-forming peptide (KLVFF). Along with the formation of this nanostructure, photothermal conversion efficiency, ROS generation, as well as retention time on the tumor were greatly improved, which enhanced both the photothermal and photodynamic effects. This superior in situ receptor-mediated strategy allowed us to achieve the goal of a single injection for irradiation.

## Supplementary Material

Supplemental Material
