# Supplementary material for: Phototherapeutic effect of transformable peptides containing pheophorbide a on colorectal cancer
Source: Drug Deliv. 2022 May 25;29(1):1608–19. doi: 10.1080/10717544.2022.2075987 (PMC9135428; doi:10.1080/10717544.2022.2075987)
Supplement: Supplemental Material [file IDRD_A_2075987_SM7204.docx]

**Supplementary Materials**

Phototherapeutic effect of transformable peptides containing Pheophorbide A on colorectal cancer

Zhiqin Zhang^a^, Kaixin Wang^a^, Manting Liu^a^, Panxiang Hu^a^, Yuchen Xu^a^, Dongge Yin^a^, Yuchang Yang^a^, Xiaoxv Dong^a^, Changhai Qu^a^, Lu Zhang ^b,^*, Jian Ni^a,^*, and Xingbin Yin^a,^*

*^a^School of Chinese Material Medical, Beijing University of Chinese Medicine, Beijing, 102488, China; ^b^Department of Biomedical Engineering, Southern University of Science and Technology, Guangdong, Shenzhen, 518055, China*

**
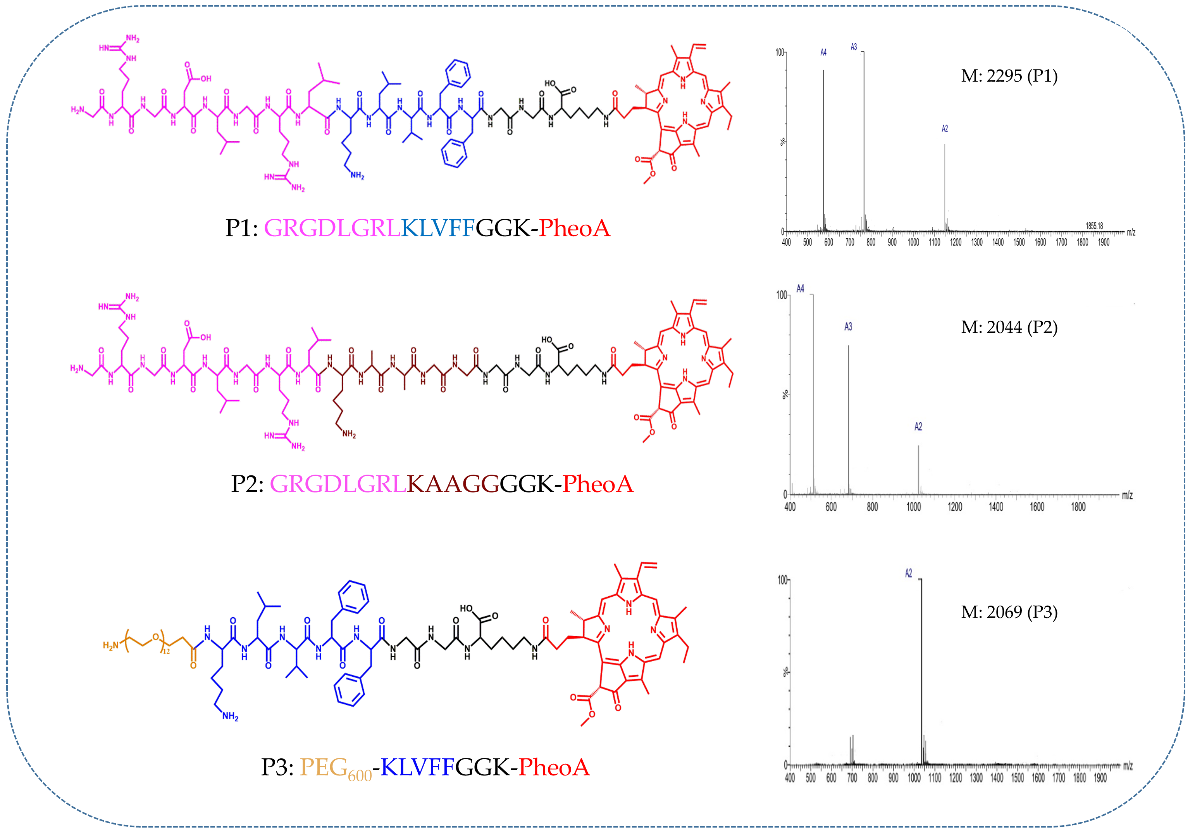
**

**Figure S1.** Chemical structure and ESI mass spectrometry of three peptide monomers GRGDLGRL-KLVFF-GGK-PheoA (P1), GRGDLGRL-KAAGG-GGK-PheoA (P2) and PEG_600_-KLVFF-GGK-PheoA.


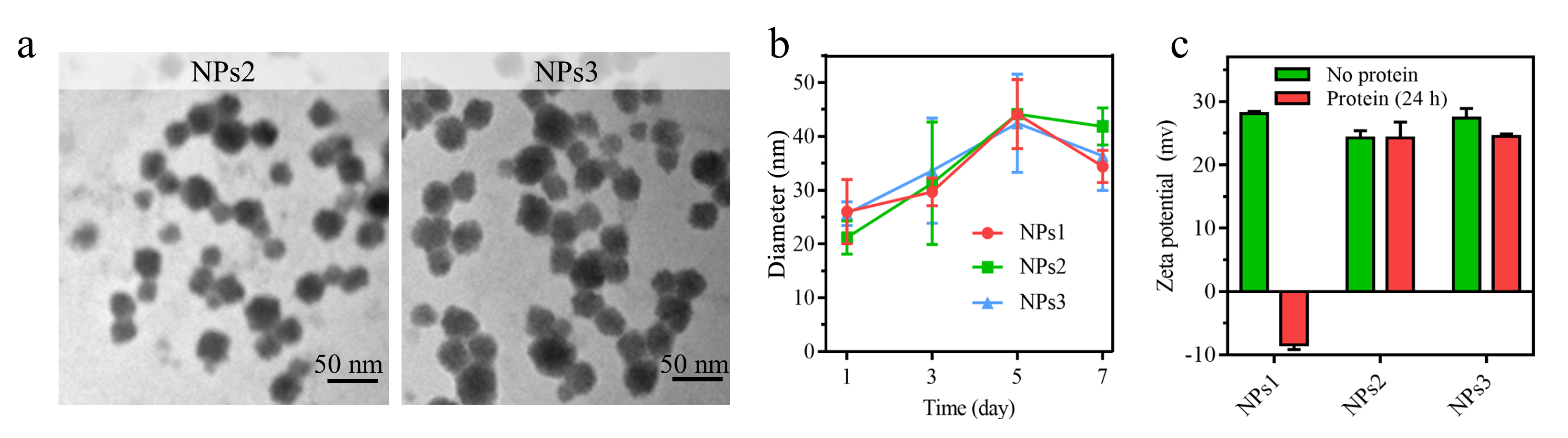


**Figure S2.** (a) TEM images of fresh NPs2 and NPs3 at the water and DMSO ratio of 995:5. (b) Size distribution of different NPs in PBS within seven days. (c) Zeta potential variation of different NPs with/without αvβ6 protein incubation for 24 h at the molar ratio of 500:1. The concentration of NPs related to these experiments was 20 μM, and all these experiments were repeated three times.


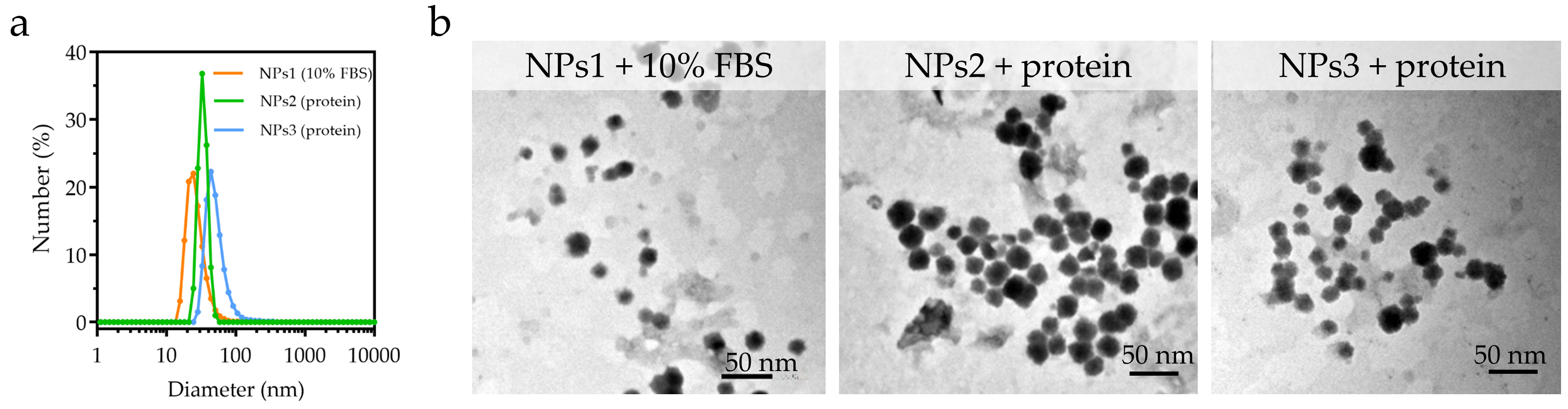


**Figure S3.** Size distribution variation (a) and TEM images (b) of NPs1 (treatment with 10% FBS for 24 h) and positive control groups NPs2 and NPs3 (incubation with αvβ6 protein for 24 h). The concentration of NPs related to these experiments was 20 μM, and all these experiments were repeated three times.

**
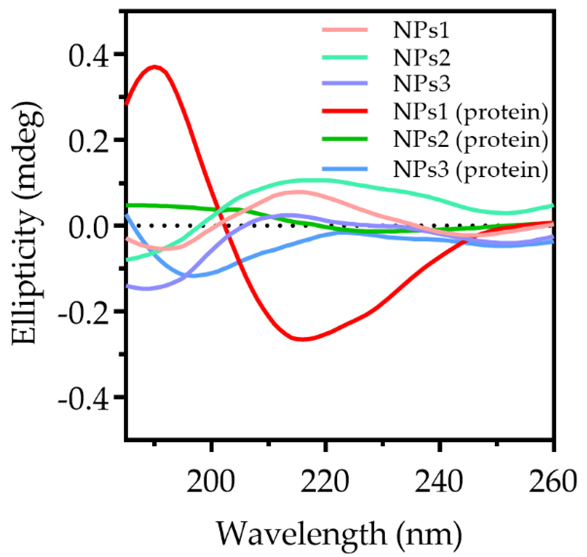
**

**Figure S4.** Circular dichroism spectra of different NPs with/without αvβ6 protein incubation for 24 h. The concentration of NPs related to these experiments was 20 μM, and all these experiments were repeated three times.


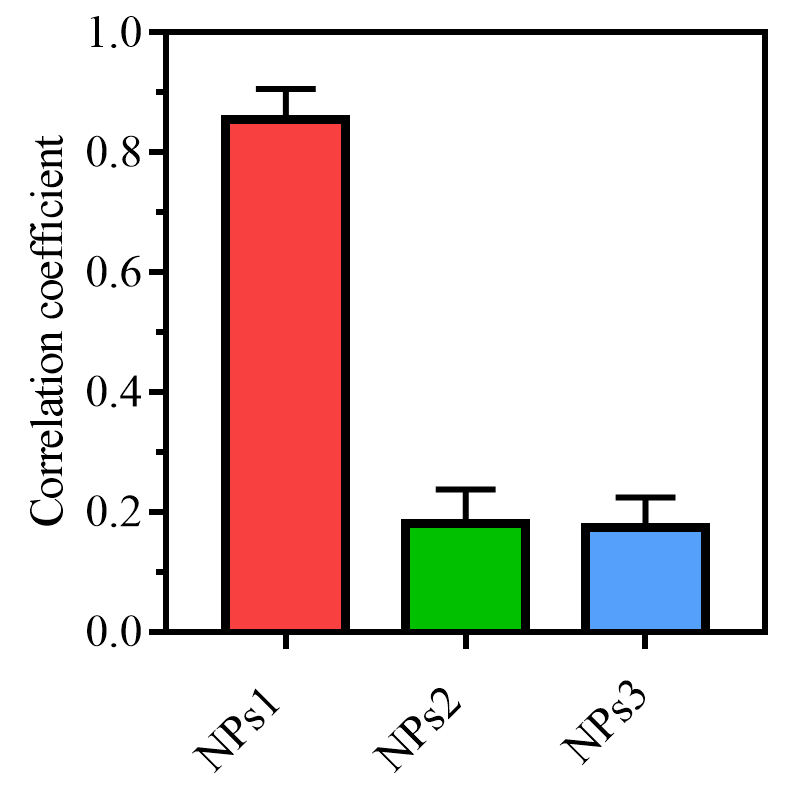


**Figure S5.** The Pearson correlation coefficient for different NPs and αvβ6 protein colocalization on the cell surface (+1, perfect correlation; -1, perfect but negative correlation; 0, the absence of a relationship).

**
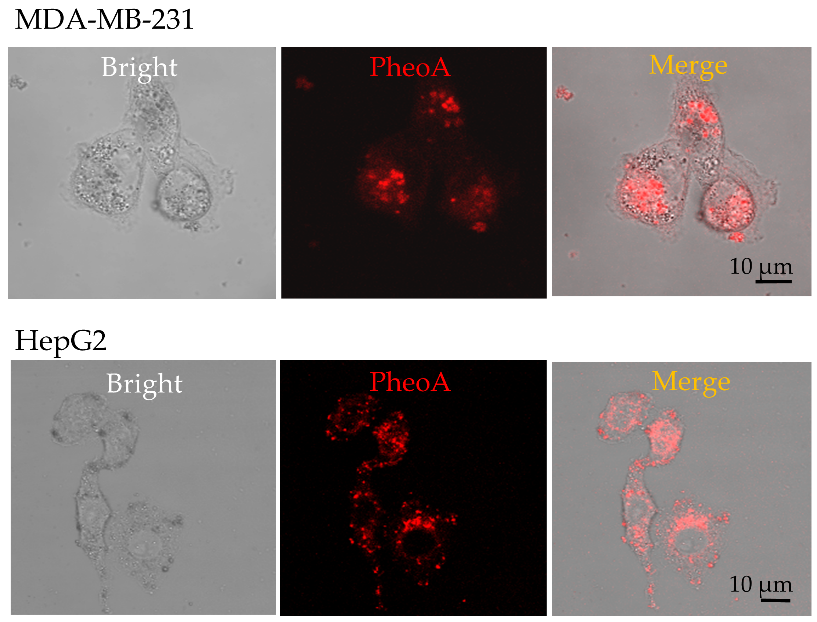
**

**Figure S6.** Fluorescence distribution images of MDA-MB-231 and HepG2 cancer cells treated with NPs1 for 8 h (excitation wavelength = 405 nm). The concentration of NPs1 related to these experiments was 20 μM, and all these experiments were repeated three times.

**
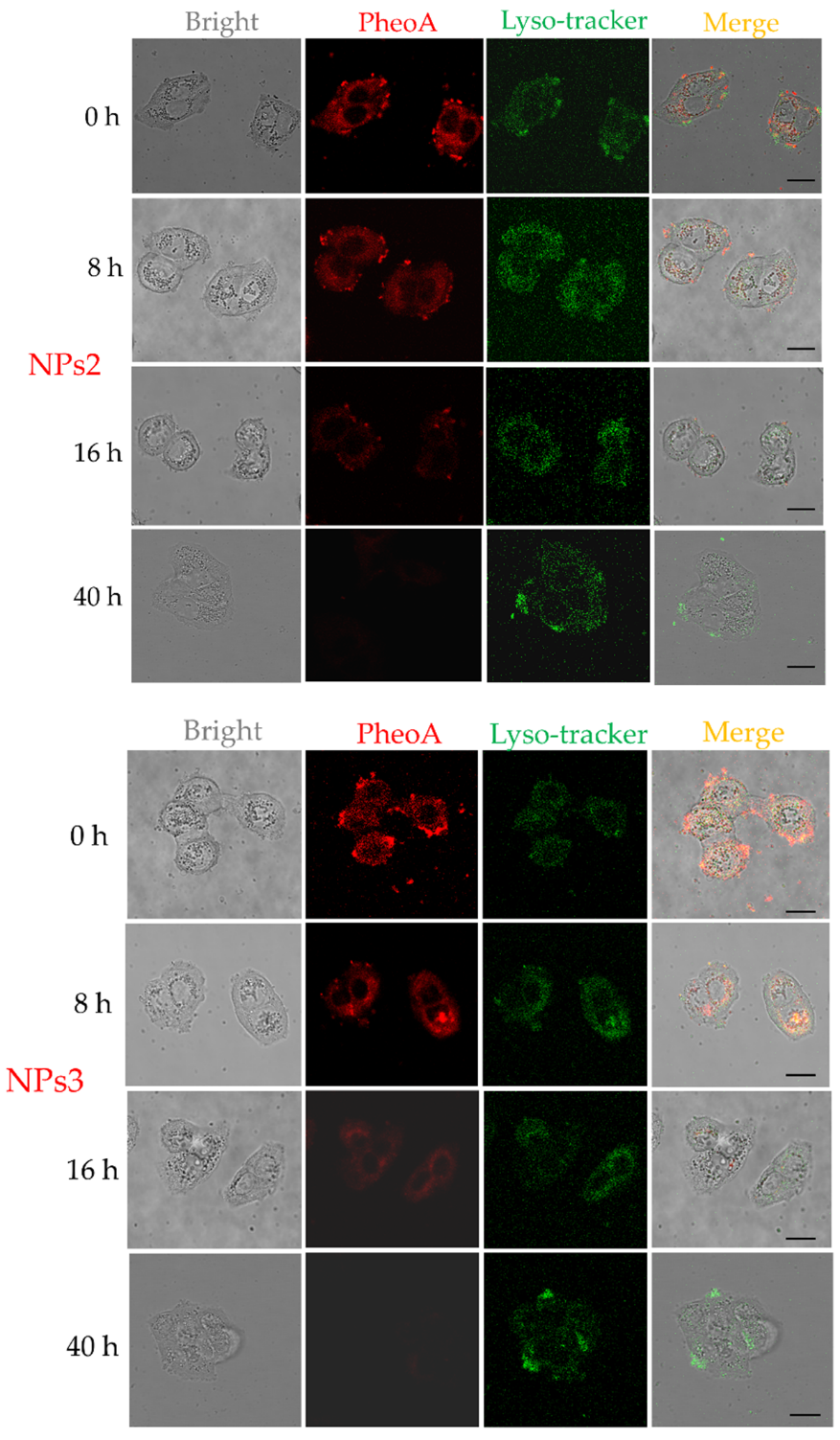
**

**Figure S7.** Variation of fluorescence distribution on tumor cells after treatment with negative control groups (PheoA: excitation wavelength = 405 nm; Lyso-tracker: excitation wavelength = 488 nm). Cancer cells were incubated with NPs2 (20 μM) and NPs3 (20 μM) for 8 h, respectively, and then the complete DMEM was removed and replaced with flesh complete DMEM without NPs for another 8, 16 and 40 h. After that, green color lysosome-tracker was added to the complete DMEM (50 nM), and was incubated with cells for 30 min. The scale bar was 10 μm.


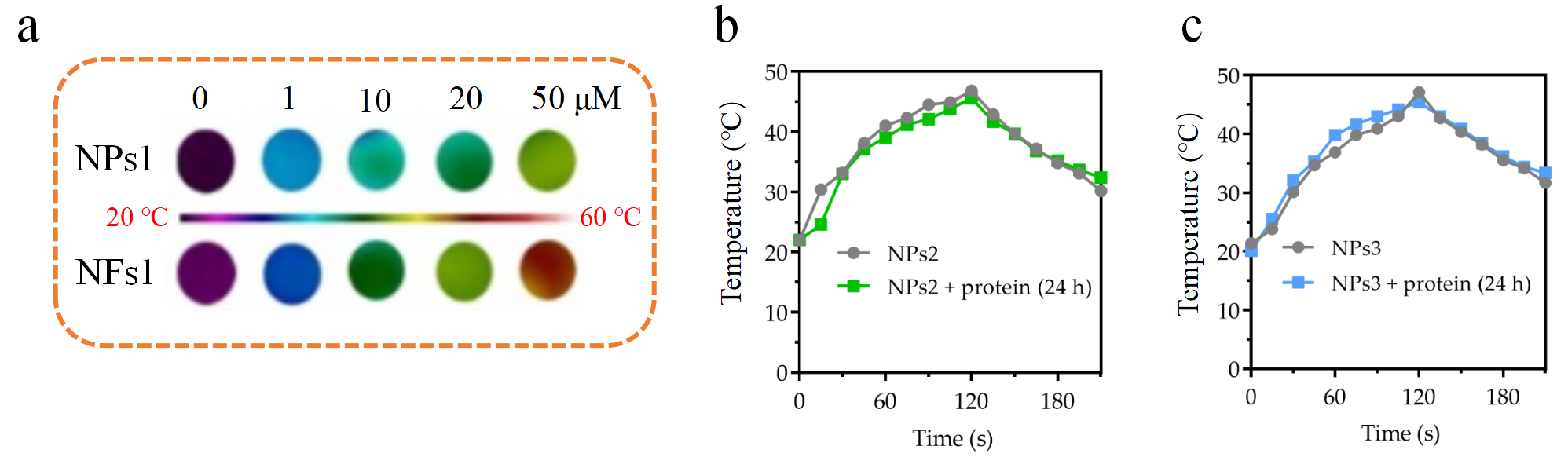


**Figure S8.** Thermal imaging of NPs1 and NFs1 at different concentration after irradiation for 120 s (0.4 W/cm^2^). Variation in temperature of NPs2 (b) and NPs3 (c) with/without αvβ6 protein incubation for 24 h after irradiation (0.4 W/cm^2^).

**
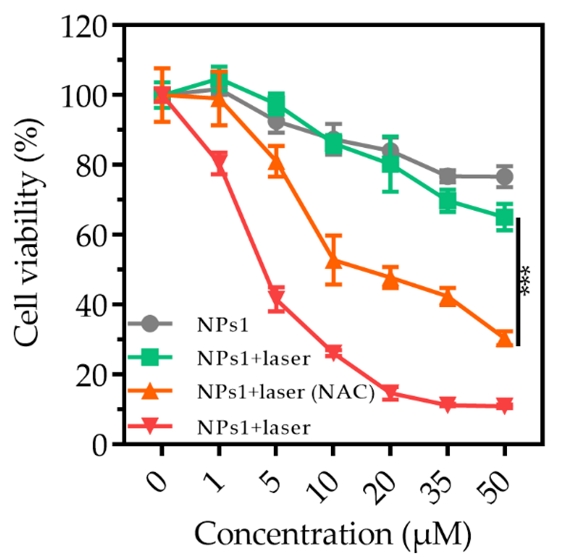
**

**Figure S9.** The cell viability of HT-29 cancer cells treatment with NPs1 in different conditions.


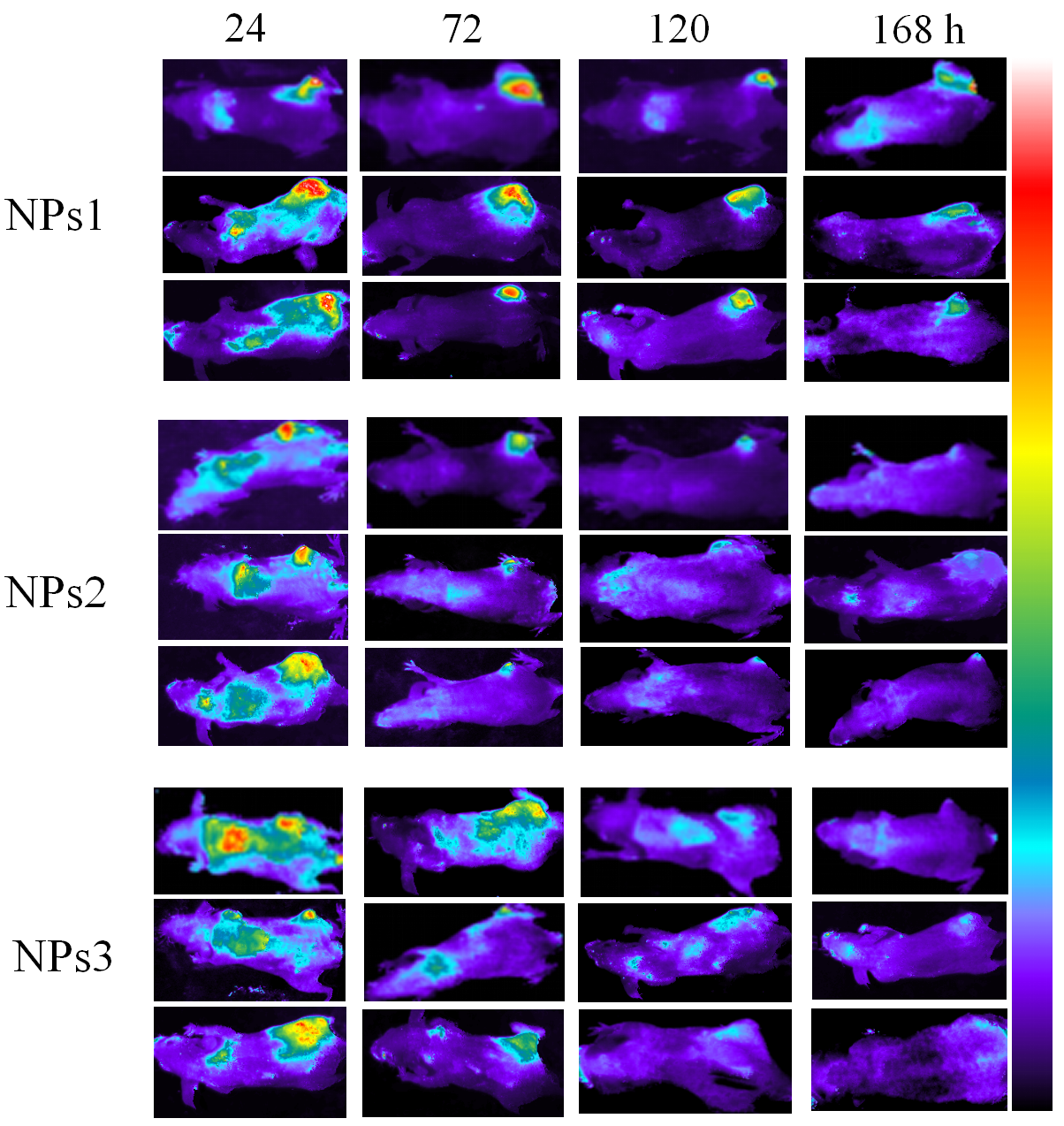


**Figure S10.** Whole fluorescence distribution images of HT-29 tumor bearing mice at different time point including 24, 72, 120, and 168 h, after treatment with different NPs (n = 3, excitation wavelength = 405 nm).


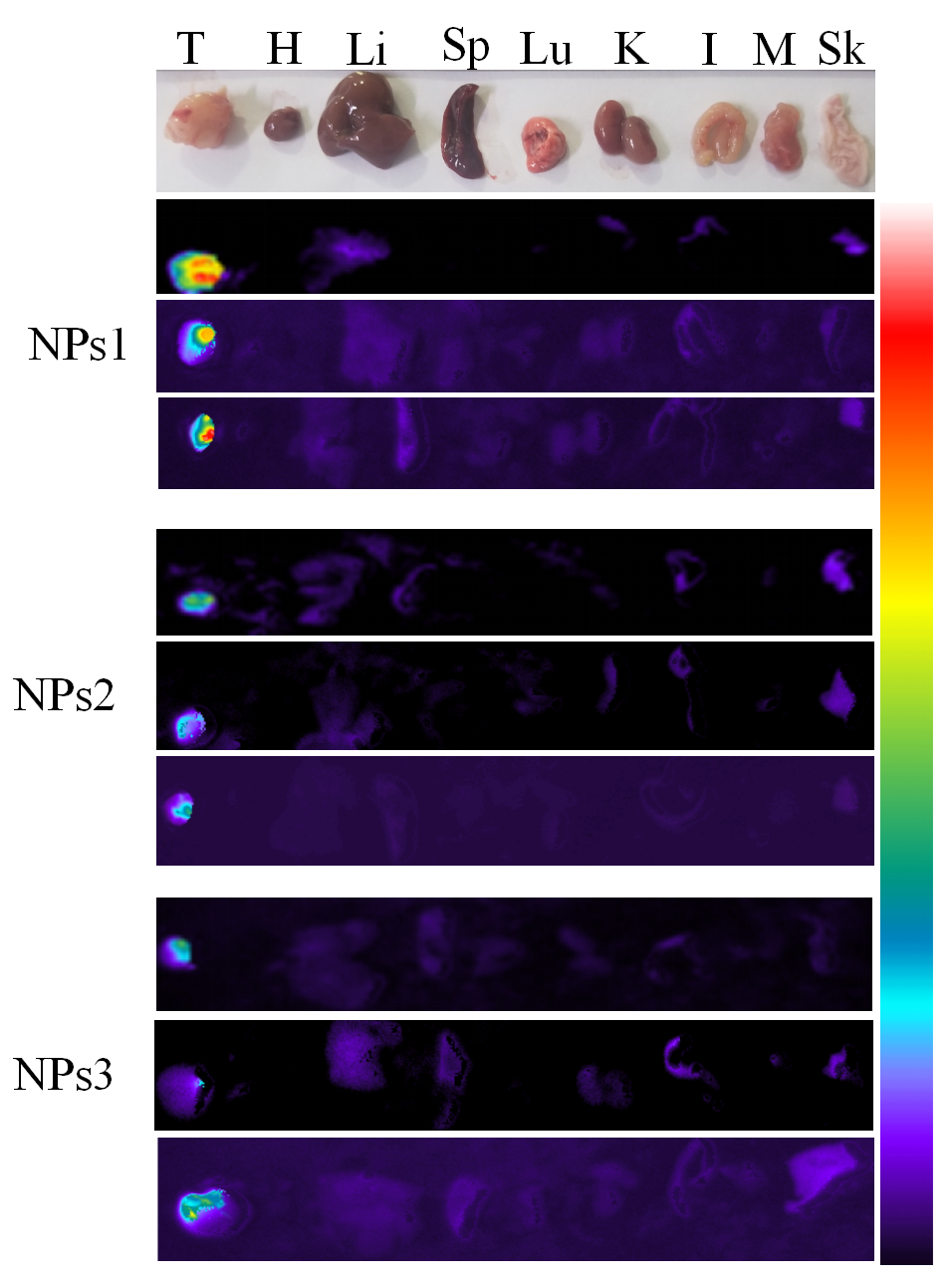


**Figure S11.** Excised tumors and several organs fluorescence imaging after i.v. injection with different NPs for 168 h (n = 3; excitation wavelength = 405 nm; T, H, Li, Sp, Lu, K, I, M, and Sk represented tumor, heart, liver, spleen, lung, kidney, intestine, muscle, and skin, respectively).
